# Secobutanolides Isolated from *Lindera obtusiloba* Stem and Their Anti-Inflammatory Activity

**DOI:** 10.3390/molecules29184292

**Published:** 2024-09-10

**Authors:** Hye Jin Yang, Young-Sang Koh, MinKyun Na, Wei Li

**Affiliations:** 1 College of Pharmacy, Chungnam National University, Daejeon 305-764, Republic of Korea; hjyang@kiom.re.kr; 2 Korean Medicine (KM) Application Center, Korea Institute of Oriental Medicine, Daegu 41062, Republic of Korea; 3 School of Medicine and Brain Korea 21 PLUS Program, Institute of Medical Science, Jeju National University, Jeju 690-756, Republic of Korea; yskoh7@jejunu.ac.kr

**Keywords:** *Lindera obtusiloba*, Lauraceae, secobutanolide, anti-inflammatory activity

## Abstract

In this study, a new secobutanolide, named secosubamolide B (**3**), along with three previously known secobutanolides (**1**, **2**, and **4**), were successfully isolated from a methanol extract of the stem of *Lindera obtusiloba*. The chemical structures of these compounds were elucidated through the analysis of spectroscopic data, and then compared with the existing literature to confirm their identities. Furthermore, the anti-inflammatory effect of these isolated compounds on bone marrow-derived dendritic cells stimulated by lipopolysaccharide (LPS) was evaluated. Compounds **1**–**3** showed the significant suppression of LPS-triggered IL-6 and IL-12 p40 production, with IC_50_ values between 1.8 and 24.1 µM. These findings may provide a scientific foundation for developing anti-inflammatory agents from *L. obtusiloba*.

## 1. Introduction

Traditional Oriental medicine presents an abundant source of potential therapeutic agents that have yet to be fully explored by Western medicine. In Korea, decoctions prepared from the wood and bark of *Lindera obtusiloba* (Lauraceae) have been traditionally utilized for their anti-inflammatory properties, as well as for enhancing blood circulation and treating skin conditions such as dermatitis [1,2,3]. Additionally, herbal infusions made from *L. obtusiloba* have been historically used in the management of chronic liver diseases. The plant’s broad medicinal applications are largely due to its diverse range of bioactive compounds. Among the bioactive components identified in the leaves of *L. obtusiloba* are lignans and butanolides, which have been shown to possess significant anti-tumor, anti-allergic, and anti-inflammatory effects. Further research has also highlighted the plant’s antioxidant, hepatoprotective, and anti-coagulant properties, underscoring its considerable therapeutic potential [4,5,6]. In particular, secobutanolides from genus Lindera have shown promising anticancer effects against HepG2, P-388, A549 and HT-29 cancer cell lines, and also shown collagen-induced platelet aggregation inhibitory activity [7,8,9].

In our recent study, we set out to isolate and identify a novel butanolide that could expand the known spectrum of active compounds derived from *L. obtusiloba*. Through our investigation, we successfully isolated and elucidated the chemical structure of a new secobutanolide, in addition to three previously known secobutanolides, from a methanol extract of the plant’s stem. Moreover, we explored the anti-inflammatory potential of these isolated compounds by assessing their effects on lipopolysaccharide (LPS)-stimulated bone marrow-derived dendritic cells (BMDCs).

Lipopolysaccharide (LPS), a predominant component of the outer membrane of gram-negative bacteria, is known for its ability to suppress inflammatory responses in vivo. However, LPS is also capable of inducing the release of various inflammatory cytokines in different cell types, leading to a robust acute inflammatory response to pathogens. Among the cytokines released, interleukin (IL)-6, IL-12, and tumor necrosis factor (TNF)-α are particularly important as key mediators of inflammation. IL-6 is especially noteworthy due to its dual role in promoting and regulating inflammation, making it a central player in many inflammatory diseases. IL-12 is crucial in the initiation and regulation of the cellular immune response, with its p40 and p80 subunits exhibiting distinct biological activities, independent of IL-12 and IL-23. Dendritic cells (DCs) are essential in orchestrating immune responses, primarily through their ability to modulate T-cell functions [10,11,12,13]. To uncover the anti-inflammatory components of *L. obtusiloba*, we tested the isolated compounds for their ability to modulate the LPS-induced expression of the pro-inflammatory cytokines IL-6 and IL-12 p40 in BMDCs. This investigation has contributed to a deeper understanding of the anti-inflammatory potential of compounds derived from *L. obtusiloba*, further supporting its use in traditional medicine and its potential use in therapeutic applications.

## 2. Results and Discussion

### 2.1. Isolation and Structural Characterization

**Figure 1:**
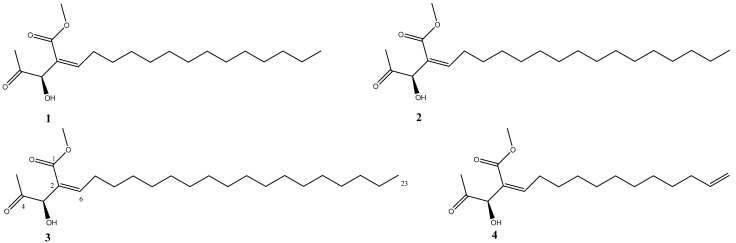
Structures of compounds **1**–**4** from the stem of *L. obtusiloba*.

Four secobutanolides were successfully isolated from a MeOH extract of *L. obtusiloba* stems. These compounds were identified as secomahubanolide (**1**) [14], secosubamolide A (**2**) [15], secosubamolide B (**3**), and secoisolitsealiicolide B (**4**) [16] (Figure 1). Notably, secosubamolide B (**3**) is a newly discovered compound, and all four compounds were identified in *L. obtusiloba* for the first time. This study represents the first thorough chemical investigation of secobutanolides in *L. obtusiloba*.

Compound **1** was isolated as a yellow oil. High-resolution electrospray ionization time-of-flight mass spectrometry (HR-ESI-TOF-MS) established its molecular formula as C_20_H_36_O_4_, with an observed *m*/*z* of 341.2690 ([M + H]^+^). The ^1^H-NMR spectrum of compound **1** (Table 1) displayed three characteristic signals: an olefinic proton at δ_H_ 7.08 (1H, t, *J* = 7.6 Hz), an oxymethine proton at δ_H_ 4.90 (1H, d, *J* = 5.0 Hz), and a methoxy group at δ_H_ 3.73 (3H, s). Correspondingly, the ^13^C-NMR spectrum (Table 1) revealed eight signals, including quaternary carbon atoms of a double bond at δ_C_ 129.7 (C-2) and 149.1 (C-6), a ketone group at δ_C_ 206.4 (C-4), and a carboxyl group at δ_C_ 166.6 (C-1), consistent with the structure of secomahubanolide, as reported by Cheng in 2005. These data indicated the presence of a secobutanolide skeleton. Compound **1** exhibited the *E*-form geometry of the trisubstituted double bond [δ_H_ 7.08 (1H, t, *J* = 7.6 Hz)] and demonstrated optical activity with ${\left[\alpha \right]}_{D}^{25}$: −13.1° (c 0.32, CHCl_3_), confirming a 3*R* configuration [15,16]. Based on these observations, the structure of compound **1** was identified as secomahubanolide.

Compound **2** was obtained as a yellow oil. The molecular formula was established as C_22_H_40_O_4_ by high-resolution electrospray ionization time-of-flight mass spectrometry (HR-ESI-TOF-MS; *m*/*z* 369.2996 ([M + H]^+^)). Both the ^1^H-NMR spectrum [δ_H_ 6.91 (1H, t, *J* = 7.2 Hz), δ_H_ 4.84 (1H, d, *J* = 5.0 Hz), and δ_H_ 3.62 (3H, s)] and ^13^C-NMR spectrum [δ_C_ 132.2 (C-2), 147.2 (C-6), 166.2 (C-1), and 208.8 (C-4)] were similar to that of secomahubanolide (**1**) (Table 1). Compound **2** exhibited the *E*-form geometry of the trisubstituted double bond, as indicated by the ^1^H NMR signal at δ_H_ 6.91 (1H, t, *J* = 7.6 Hz). Its optical rotation was measured at ${\left[\alpha \right]}_{D}^{25}$: −11.7° (c 0.15, CHCl_3_), suggesting a 3*R* configuration [15,16]. Additionally, compound **2** featured two extra methylene units at δ_H_ 1.24 in its side chain compared to secomahubanolide (**1**). Based on these characteristics, the structure of compound **2** was determined to be secosubamolide A.

Compound **3** was also isolated as a yellow oil, with its molecular formula established as C_24_H_44_O_4_ through high-resolution electrospray ionization time-of-flight mass spectrometry (HR-ESI-TOF-MS), yielding an *m*/*z* of 397.3319 ([M + H]^+^). The HR-MS spectrum clearly shows that compound **3** has two more methylene groups than compound **2**, just as compound **2** has two more methylene groups than compound **1**. The HR-MS data for all three compounds exhibit a consistent increasing trend. Therefore, we can conclude that compound **3** is compound **2** with two additional methylene groups on the side chain, and this inference is further confirmed by the NMR spectra. The ^1^H NMR (Table 1) spectrum revealed signals at δ_H_ 7.07 (1H, t, *J* = 7.2 Hz), δ_H_ 4.89 (1H, d, *J* = 5.0 Hz), and δ_H_ 3.72 (3H, s), while the ^13^C NMR spectrum showed resonances at δ_C_ 132.0 (C-2), 146.8 (C-6), 166.2 (C-1), and 208.9 (C-4), closely resembling those of secomahubanolide (**1**) and secosubamolide A (**2**). Compound **3** maintained the *E*-form geometry of the trisubstituted double bond, as seen in the signal at δ_H_ 6.82 (1H, t, *J* = 7.6 Hz), and exhibited an optical rotation of ${\left[\alpha \right]}_{D}^{25}$: −21.7° (c 0.52, CHCl_3_), confirming the 3*R* configuration [15,16]. Moreover, compound **3** displayed two additional methylene units at δ_H_ 1.24 in its side chain compared to secosubamolide A (**2**). Based on these observations, the structure of compound **3** was identified as secosubamolide B.

### 2.2. Anti-Inflammatory Activity of Compounds ***1***–***3***

Prior to evaluating the anti-inflammatory potential of secobutanolides isolated from the methanol extract of *L. obtusiloba*, we initially examined the effects of compounds **1**–**3** (at a 50 μM concentration) on BMDC cell viability using an MTT colorimetric assay. The results showed that these compounds did not exhibit any cytotoxicity at the concentrations tested (data not shown).

**Figure 2:**
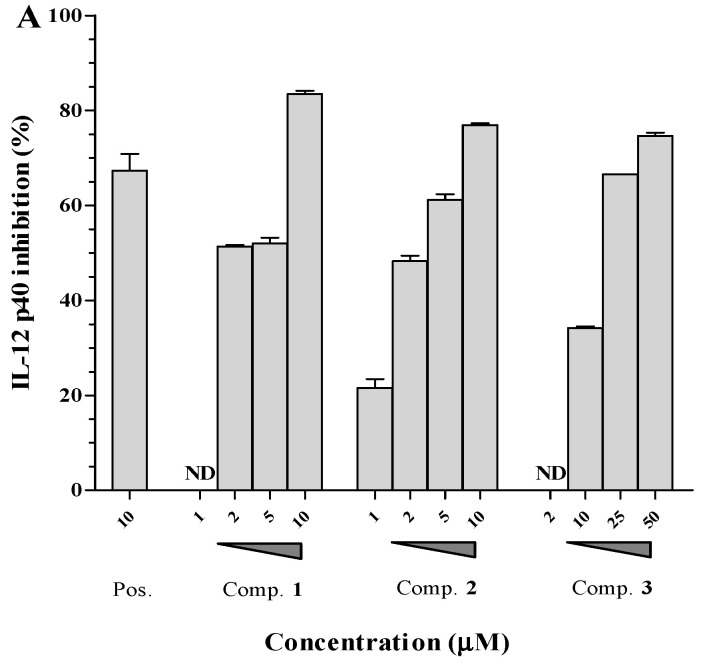

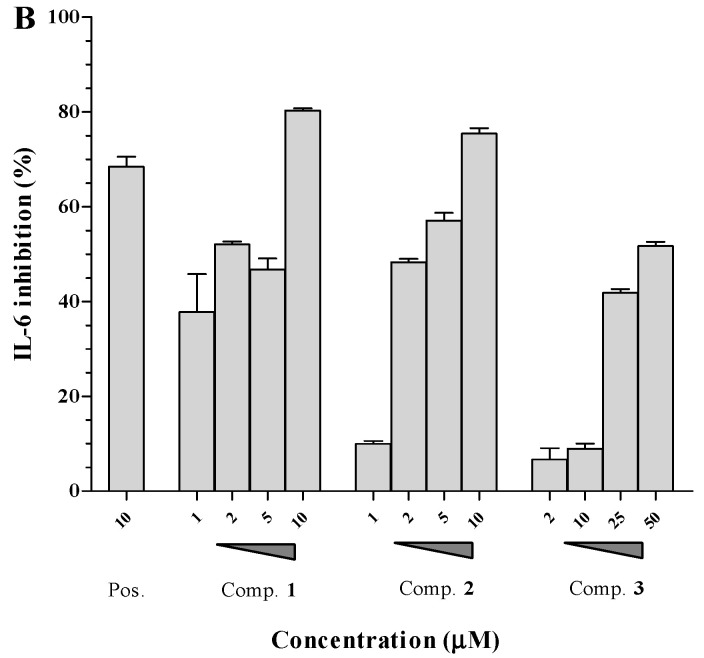
Effect of compounds **1**–**3** on the production of IL-12 p40 (**A**) and IL-6 (**B**) in LPS-stimulated BMDCs at concentrations of 1.0–50.0 μM. The results are shown as inhibition rates (%) relative to the levels observed in vehicle-treated DCs. Pos: SB203580. ND = Not Detected.

Following this, the effects of compounds **1**–**3** on the production of IL-6 and IL-12 p40 were assessed at various concentrations ranging from 1 to 50 μM. In these experiments, 4-(4-Fluorophenyl)-2-(4-methylsulfinylphenyl)-5-(4-pyridyl)-1H-imidazole (SB203580) was utilized as a positive control [14]. SB203580 demonstrated a marked ability to inhibit the production of both IL-6 and IL-12 p40, with calculated IC_50_ values of 3.5 μM and 5.0 μM, respectively (Figure 2). The tested secobutanolides, identified as compounds **1**–**3**, also showed significant inhibitory effects on IL-6 production, with IC_50_ values determined to be 1.8 μM, 3.4 μM, and 24.1 μM, respectively. Similarly, these compounds exerted strong inhibitory activity against IL-12 p40 production, with IC_50_ values of 3.1 μM, 2.9 μM, and 16.3 μM, as shown in Table 2.

This study not only highlights the robust anti-inflammatory properties of these secobutanolides, but also provides important insights into their structure–activity relationships. Notably, the inhibitory effects were significantly amplified by the presence of unsaturated aliphatic substituents on the acylglycerol framework. Moreover, the activity of these compounds appeared to increase proportionally with the length of the fatty acid side chains, indicating that these structural characteristics are critical for their bioactivity. These findings could be pivotal in informing the design and synthesis of new anti-inflammatory agents derived from the secobutanolide structure, offering a promising direction for future drug development efforts.

## 3. Materials and Methods

### 3.1. General Information

Column chromatography: silica gel (Kieselgel 60, 70−230, and 230−400 mesh, Merck, Darmstadt, Germany); YMC RP-18 resins, and thin-layer chromatography (TLC): silica-gel 60 F_254_ and RP-18 F_254_S plates (both 0.25 mm, Merck, Darmstadt, Germany); optical rotation: Jasco DIP-370 automatic polarimeter (Easton, MD, USA); NMR spectra: JEOL ECA 600 spectrometer (Tokyo, Japan); High-resolution electrospray ionization mass spectra (HR-MS): Agilent 6530 Accurate-Mass Q-TOF LC/MS system (Santa Clara, CA, USA). 

### 3.2. Plant Material

In 2001, dried stems of *L. obtusiloba* were provided by Bomyeong Herbal Market (Seoul, Republic of Korea), Korea, and the voucher specimen (KM-000101) was deposited with the Korean Medicine (KM) Application Center, Korea Institute of Oriental Medicine, Korea.

### 3.3. Extraction and Isolation

The air-dried and chopped stems of *L. obtusiloba* (3.0 kg) were extracted three times with MeOH for 10 h in a water bath. The MeOH extract obtained was evaporated to dryness in a rotary evaporator, affording crude extract (185.3 g, 6.1% yield). The extract was dissolved in distilled water (1.5 L), and 1.2 L of the resulting solution was consecutively partitioned using CHCl_3_, EtOAc, and *n*-BuOH; CHCl_3_ (48.5 g), EtOAc (20.4 g), *n*-BuOH (36.8g), and H_2_O fractions were obtained.

A portion of the CHCl_3_ layer (48.5 g) was subjected to column chromatography using silica gel column chromatography and eluted with a stepwise gradient of EtOAc:MeOH (20:1–10:1–5:1–2:1–1:1, *v*/*v*) to yield three fractions (1a–c). Fraction 1a was chromatographed on a silica gel column using a gradient of *n*-Hexane:EtOAc (8:1–3:1 *v*/*v*) to afford sub-fractions (2a–c). Fraction 2b was further chromatographed on a silica gel chromatography column with CHCl_3_:MeOH (90:1–70:1 *v*/*v*) to afford subfractions (3a–b). Fraction 3d was further chromatographed on a silica gel chromatography column with CHCl_3_:MeOH (60:1 *v*/*v*) to yield compound **1** (12.1 mg). Fraction 3b was chromatographed on a silica gel column *n*-Hexane:acetone (8:1–7:1 *v*/*v*) to give compounds **2** (24.1 mg), **3** (2.4 mg), and **4** (49.9 mg). 

Secomahubanolide (**1**): yellow oil; UV (MeCN): λ_max_ 215 nm; HR-ESI-TOF-MS *m*/*z* 341.2690 [M + H]^+^; ${\left[\alpha \right]}_{D}^{25}$: −13.1° (c = 0.32, CHCl_3_); IR (KBr, cm^−1^) 3450, 1735, 1710 cm^−1^; ^1^H-NMR (600 MHz) and ^13^C-NMR (150 MHz): see Table 1.

Secosubamolide A (**2**): yellow oil; UV (MeCN): λ_max_ 215 nm; HR-ESI-TOF-MS *m*/*z* 369.2996 [M + H]^+^; ${\left[\alpha \right]}_{D}^{25}$: −11.7° (c = 0.15, CHCl_3_); IR (KBr, cm^−1^) 3450, 1735, 1710 cm^−1^; ^1^H-NMR (600 MHz) and ^13^C-NMR (150 MHz): see Table 1.

Secosubamolide B (**3**): yellow oil; UV (MeCN): λ_max_ 215 nm; HR-ESI-TOF-MS *m*/*z* 397.3319 [M + H]^+^; ${\left[\alpha \right]}_{D}^{25}$: −21.7° (c = 0.52, CHCl_3_); IR (KBr, cm^−1^) 3450, 1735, 1710 cm^−1^; ^1^H-NMR (600 MHz) and ^13^C-NMR (150 MHz): see Table 1. HR-MS, UV, and NMR data of compound **3** can be found in the Supplementary Materials.

Secoisolitsealiicolide B (**4**): yellow oil; UV (MeOH): λ_max_ 213 nm; ^1^H-NMR (400 MHz, CDCl_3_, δ_H_): 7.08 (1H, t, *J* = 7.5, H-6), 5.81 (1H, ddt, *J* = 17.5, 10.6, 6.8 Hz, H-16), 4.99 (1H, dd, *J* = 17.2, 2.1 Hz, H-17a), 4.93 (1H, dd, *J* = 10.3, 1.4 Hz, H-17b), 4.90 (1H, s, H-3), 4.03 (1H, s, 3-OH), 3.73 (3H, s, 1-OCH_3_), 2.35 (2H, quartet, *J* = 7.6 Hz, H-7), 2.16 (3H, s, H-5), 2.04 (2H, quartet, *J* = 6.9 Hz, H-15), 1.28 (2H, br s, H-9), 1.28 (2H, br s, H-10), 1.28 (2H, br s, H-11), 1.28 (2H, br s, H-12), 1.28 (2H, br s, H-13), 1.28 (2H, br s, H-14); ^13^C-NMR (100 MHz, CDCl_3_, δ_C_): 206.5 (C-4), 166.6 (C-1), 149.2 (C-6), 139.2 (C-16), 129.8 (C-2), 114.2 (C-17), 73.4 (C-3), 52.0 (1-OCH_3_), 33.7 (C-15), 28.8 (C-7), 28.6 (C-8), 28.7–29.5 (C-9), 28.7–29.5 (C-10), 28.7–29.5 (C-11), 28.7–29.5 (C-12), 28.7–29.5 (C-13), 28.7–29.5 (C-14), 24.9 (C-5).

### 3.4. Cell Culture

The BMDCs were derived from wild-type C57BL/6 mice. Briefly, bone marrow cells were cultured in RPMI 1640 medium containing 10% heat-inactivated fetal bovine serum (FBS), 50 μM of 2-ME, and 2 mM of glutamine supplemented with 3% J558L hybridoma cell culture supernatant containing granulocyte macrophage colony-stimulating factor for dendritic cell generation. The culture medium was replaced with fresh medium every second day. On day 6 of culture, non-adherent cells and loosely adherent DC aggregates were harvested, washed and resuspended in RPMI 1640 supplemented with 5% FBS. All animal procedures were approved and performed according to the guidelines of the Institutional Animal Care and Use Committee of Jeju National University (#2010−0028).

### 3.5. Cytokine Production Measurements

The effect of isolated compounds on cytokine production by LPS-stimulated cells was determined using ELISA (BD PharMingen, San Diego, CA, USA). The final concentration of chemical solvents did not exceed 0.1%. BMDCs were cultured in DMEM medium containing macrophage colony-stimulating factor. On day 6 of incubation, BMDMs and BMDCs were harvested and seeded in 48-well plates at a density of 1 × 10^5^ cells/0.5 mL, and then treated with the PTE for 1 h before stimulation with LPS (10 ng/mL). At the end of the incubation period, the IL-12 p40 and IL-6 levels in the medium were determined by ELISA. All experiments were performed in triplicate.

### 3.6. Statistical Analysis

The student’s t-test and one-way ANOVA were used to determine the statistical significance of the differences between values for a variety of experimental and control groups. Data were expressed as the mean ± SD (at least in triplicate). The statistical significance was determined by one-way analysis of variance followed by Dunnett’s multiple comparison tests, *p* < 0.05. 

## 4. Conclusions

In this study, four compounds, including a newly discovered secobutanolide (compound **3**), were successfully isolated from a methanol extract of *Lindera obtusiloba*. The inhibitory effects of the isolated compounds (**1**–**3**) were assessed in terms of their ability to suppress the production of cytokines IL-12p40 and IL-6, which are stimulated by lipopolysaccharide (LPS) in bone marrow-derived dendritic cells (BMDCs). Notably, this research represents the first investigation into the secobutanolide components of *L. obtusiloba* and their potential anti-inflammatory properties. The compounds isolated in this study were identified from the plant for the first time, indicating their novelty. These findings suggest that secobutanolides are active constituents of *L. obtusiloba* and may serve as a scientific foundation for developing new anti-inflammatory agents derived from this plant.

## Figures and Tables

**Table 1:** ^1^H and ^13^C NMR spectroscopic data for compounds **1**–**3** in CDCl_3_.

	1		2		3	
	δ_H_ ^a^ (*J*/Hz)	δ_C_ ^b^	δ_H_ ^a^ (*J*/Hz)	δ_C_ ^b^	δ_H_ ^a^ (*J*/Hz)	δ_C_ ^b^
1	-	166.6	-	166.2	-	166.6
2	-	129.7	-	132.2	-	129.7
3	4.90 (d, 5.0)	73.3	4.84 (d, 5.0)	72.2	4.89 (d, 5.0)	73.3
4	-	206.4	-	208.8	-	206.5
5	2.15 (s)	24.71	2.11 (s)	26.0	2.14 (s)	25.8
6	7.08 (t, 7.4)	149.1	6.91 (t, 7.2)	147.2	7.07 (t, 7.2)	149.2
7	2.35 (q, 7.4)	28.6	2.28 (q, 7.2)	28.0	2.34 (q, 7.2)	27.9
8	1.51 (m)	28.8	1.41 (m)	28.4	1.41 (m)	28.1
9	1.25 (m)	29.2	1.24 (m)	28.5–29.0	1.24 (m)	29.0–29.5
10	1.25 (m)	29.2	1.24 (m)	28.5–29.0	1.24 (m)	29.0–29.5
11	1.25 (m)	29.3	1.24 (m)	28.5–29.0	1.24 (m)	29.0–29.5
12	1.25 (m)	29.9	1.24 (m)	28.5–29.0	1.24 (m)	29.0–29.5
13	1.25 (m)	29.4	1.24 (m)	28.5–29.0	1.24 (m)	29.0–29.5
14	1.25 (m)	29.4	1.24 (m)	28.5–29.0	1.24 (m)	29.0–29.5
15	1.25 (m)	29.4	1.24 (m)	28.5–29.0	1.24 (m)	29.0–29.5
16	1.25 (m)	29.2	1.24 (m)	28.5–29.0	1.24 (m)	29.0–29.5
17	1.25 (m)	31.8	1.24 (m)	28.5–29.0	1.24 (m)	29.0–29.5
18	1.25 (m)	22.5	1.24 (m)	28.5–29.0	1.24 (m)	29.0–29.5
19	0.87 (t, 6.8)	13.9	1.24 (m)	31.5	1.24 (m)	29.0–29.5
20			1.24 (m)	22.5	1.24 (m)	29.0–29.5
21			0.86 (t, 6.7)	13.9	1.24 (m)	31.8
22					1.24 (m)	22.6
23					0.87 (t, 6.2)	13.9
1-OCH_3_	3.73 (s)	51.9	3.62 (s)	51.8	3.72 (s)	51.9

*J* values (Hz) are given in parentheses. ^a^ 600 MHz. ^b^ 150 MHz.

**Table 2:** Anti-inflammatory effects of compounds **1**–**3**.

Compound	IC_50_ (µM) ^a^	
	IL-12 p40	IL-6
**1**	3.1 ± 0.4	1.8 ± 0.1
**2**	2.9 ± 0.6	3.4 ± 0.2
**3**	16.3 ± 0.6	24.1 ± 1.2
SB203580 ^b^	5.0 ± 0.1	3.5 ± 0.2

The ^a^ IC_50_ values for selected compounds are given in column IL-12 p40 and IL-6. ^b^ Positive control.

## Data Availability

The original contributions presented in the study are included in the article/Supplementary Materials, further inquiries can be directed to the corresponding author/s.

## Supplementary Materials

The following supporting information can be downloaded at: https://www.mdpi.com/article/10.3390/molecules29184292/s1, Figure S1. HR-ESI-MS spectrum of compound 3; Figure S2. UV spectrum of compound 3 MeCN; Figure S3. ^1^H-NMR spectrum of compound 3 (600 MHz CDCl_3_); Figure S4. ^13^C-NMR spectrum of compound 3 (150 MHz CDCl_3_).

## References

[1] Kwon D.J., Kim J.K., Bae Y.S. (2007). Essential oils from leaves and twigs of Lindera obtusiloba. J. Korean For. Soc..

[2] Seo K.H., Baek M.Y., Lee D.Y., Cho J.G. (2011). Isolation of Flavonoids and Lignans from the Stem Wood of Lindera Obtusioba Blume. J. Appl. Biol. Chem..

[3] Kwon H.C., Choi S.U., Lee J.O., Bae K.H., Zee O.P., Lee K.R. (1999). Two new lignans from Lindera obtusiloba blume. Arch. Pharm. Res..

[4] Lee J.O., Oak M.H., Jung S.H., Park D.H., Auger C., Kim K.R., Lee S.W., Schini-Kerth V.B. (2011). An ethanolic extract of Lindera obtusiloba stems causes NO-mediated endothelium-dependent relaxations in rat aortic rings and prevents angiotensin II-induced hypertension and endothelial dysfunction in rats. Naunyn. Schmiedebergs. Arch. Pharmacol..

[5] Lee K.Y., Kim S.H., Jeong E.J., Park J.H., Kim S.H., Kim Y.C., Sung S.H. (2010). New secoisolariciresinol derivatives from Lindera obtusiloba stems and their neuroprotective activities. Planta Med..

[6] Bang C.Y., Choung S.Y. (2009). Antioxidant and whitening effects of Lindera obtusiloba BL. 70% EtOH extract. Planta Med..

[7] Kwon H.C., Baek N.I., Choi S.U., Lee K.R. (2000). New cytotoxic butanolides from Lindera obtusiloba BLUME. Chem. Pharm. Bull..

[8] Tsai I.L., Hung C.H., Duh C.Y., Chen I.S. (2002). Cytotoxic butanolides and secobutanolides from the stem wood of Formosan Lindera communis. Planta Med..

[9] Lin C.T., Chu F.H., Chang S.T., Chueh P.J., Su Y.C., Wu K.T., Wang S.Y. (2007). Secoaggregatalactone-A from Lindera aggregata induces apoptosis in human hepatoma hep G2 cells. Planta Med..

[10] Weinstein D.L., O’Neill B.L., Metcalf E.S. (1997). Salmonella typhi stimulation of human intestinal epithelial cells induces secretion of epithelial cell-derived interleukin-6. Infect. Immun..

[11] Mannon P.J., Fuss I.J., Mayer L., Elson C.O., Sandborn W.J., Present D., Dolin B., Goodman N., Groden C., Hornung R.L. (2004). Anti-interleukin-12 antibody for active Crohn’s disease. N. Engl. J. Med..

[12] Cooper A.M., Khader S.A. (2007). IL-12p40: An inherently agonistic cytokine. Trends Immunol..

[13] Tung N.H., Quang T.H., Son J.H., Koo J.E., Hong H.J., Koh Y.S., Song G.Y., Kim Y.H. (2011). Inhibitory effect of ginsenosides from steamed ginseng-leaves and flowers on the LPS-stimulated IL-12 production in bone marrow-derived dendritic cells. Arch. Pharm. Res..

[14] Cheng M.J., Tsai I.L., Lee S.J., Jayaprakasam B., Chen I.S. (2005). Steryl epoxide, secobutanolide and butanolides from the stem wood of Machilus zuihoensis. Phytochemistry.

[15] da Costa-Silva T.A., Conserva G.A.A., Galisteo A.J., Tempone A.G., Lago J.H.G. (2019). Antileishmanial activity and immunomodulatory effect of secosubamolide, a butanolide isolated from Nectandra oppositifolia (Lauraceae). J. Venom. Anim. Toxins. Incl. Trop. Dis..

[16] Cheng M.J., Wang T.A., Lee S.J., Chen I.S. (2010). A new butanolide and a new secobutanolide from Litsea lii var. nunkao-tahangensis. Nat. Prod. Res..

