# Supplementary material for: Secobutanolides Isolated from Lindera obtusiloba Stem and Their Anti-Inflammatory Activity
Source: Molecules. 2024 Sep 10;29(18):4292. doi: 10.3390/molecules29184292 (PMC11434359; doi:10.3390/molecules29184292)
Supplement: Supplementary file 1 [file molecules-29-04292-s001.zip › molecules-3195579-supplementary.pdf]

Supporting information

Secobutanolides Isolated from Lindera obtusiloba Stem and Their Anti-Inflammatory Activity

Hye Jin Yang 1,2, Young-Sang Koh 3, MinKyun Na 1,* and Wei Li 2,*

1 College of Pharmacy, Chungnam National University, Daejeon 305−764, Korea; hjyang@kiom.re.kr
2 Korean Medicine (KM) Application Center, Korea Institute of Oriental Medicine,
Daegu 41062, Republic of Korea
3 School of Medicine and Brain Korea 21 PLUS Program, Institute of Medical Science,
Jeju National University, Jeju 690−756, Korea; yskoh7@jejunu.ac.kr
* C orrespondence: mkna@cnu.ac.kr (M.N.); liwei1986@kiom.re.kr (W.L.)

Legends:

Figure S1. HR-ESI-MS spectrum of compound **3** --3

Figure S2. UV spectrum of compound **3** --- 3

Figure S3. ^{1}H-NMR spectrum of compound **3** (600 MHz, $CDCl_3$) ------------------------------- 4

Figure S4. ^{13}C-NMR spectrum of compound **3** (150 MHz, $CDCl_3$) ----------------------------- 4

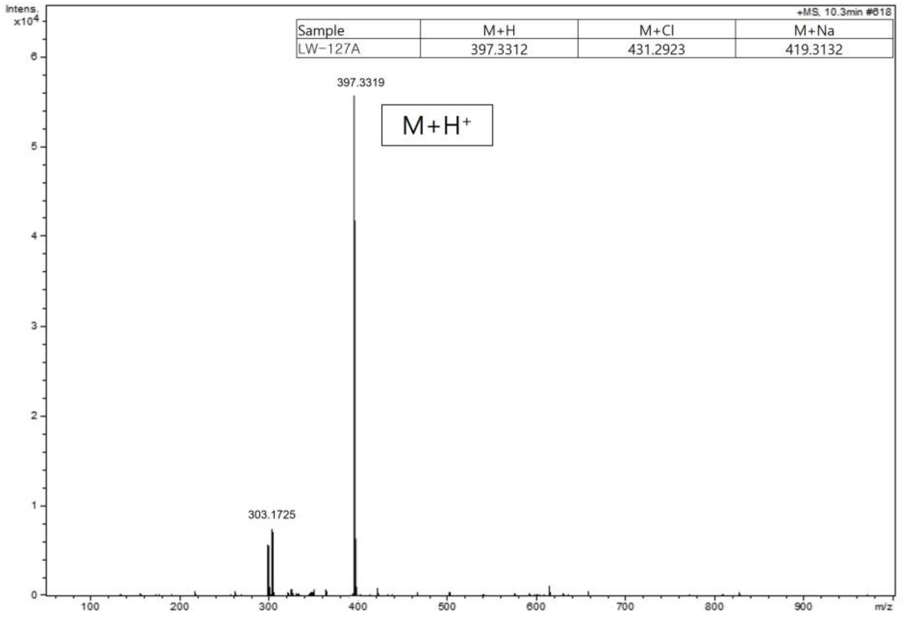

Fig. S1 HR-ESI-MS spectrum of compound **3**

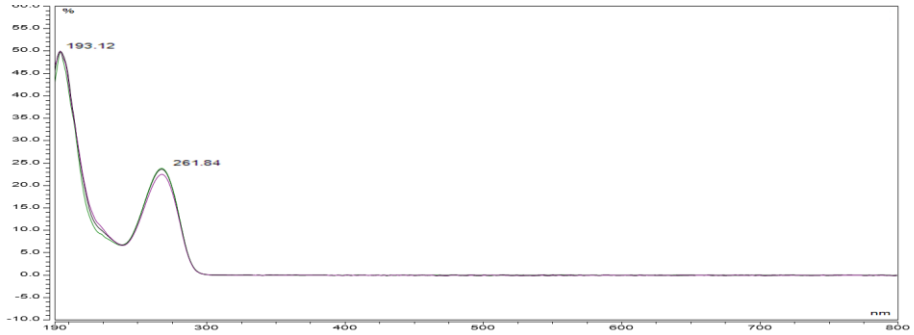

Fig. S2 UV spectrum of compound **3**

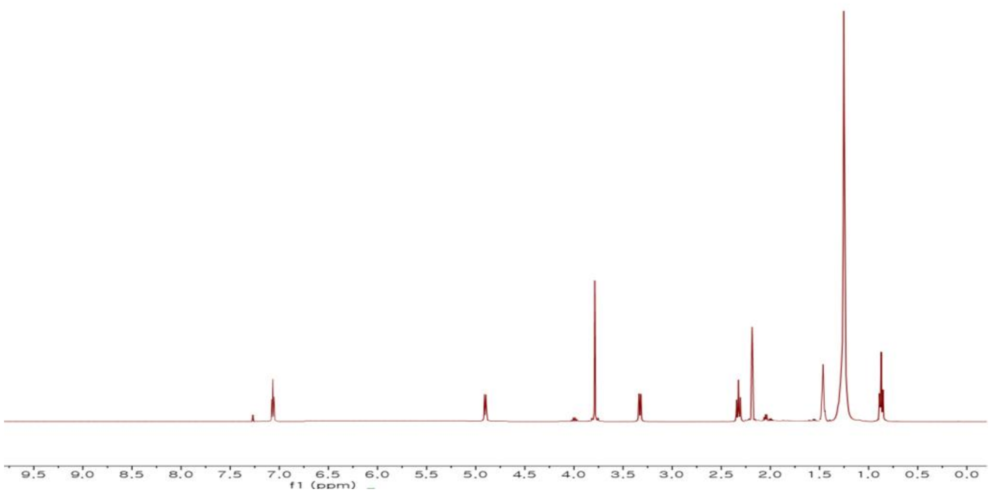

Fig. S3 ^{1}H-NMR spectrum of compound **3** (600 MHz, $CDCl_3$)

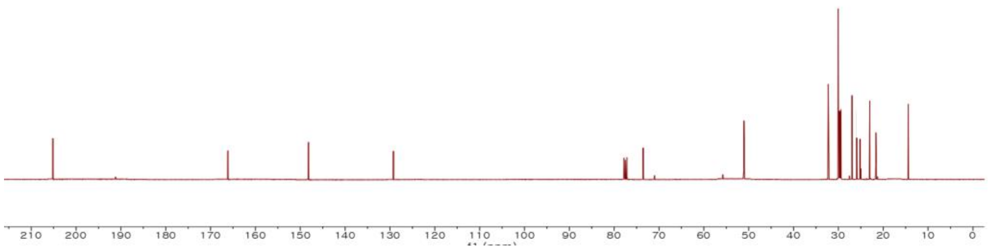

Fig. S4 ^{13}C-NMR spectrum of compound **3** (150 MHz, $CDCl_3$)
